# Novel insights into the genome organization of Rhizobiaceae: identification of linear plasmids

**DOI:** 10.1099/mgen.0.001537

**Published:** 2025-11-05

**Authors:** Nemanja Kuzmanovic, Ryan R. Wick, Michał Kalita, Joanna Puławska, George C. diCenzo, Michael F. Hynes, Kornelia Smalla, Doreen Babin, Torsten Thünen

**Affiliations:** 1 Julius Kühn Institute (JKI) - Federal Research Centre for Cultivated Plants, Institute for Plant Protection in Horticulture and Urban Green, Braunschweig, Germany; 2 Department of Microbiology and Immunology, The University of Melbourne at the Peter Doherty Institute for Infection and Immunity, Melbourne, Victoria, Australia; 3 Centre for Pathogen Genomics, The University of Melbourne, Parkville, Victoria, Australia; 4 Department of Genetics and Microbiology, Institute of Biological Sciences, Maria Curie-Skłodowska University, Lublin, Poland; 5 The National Institute of Horticultural Research, Department of Plant Protection, Skierniewice, Poland; 6 Department of Biology, Queen’s University, Kingston, Ontario, Canada; 7 Department of Biological Sciences, University of Calgary, Calgary, AB, Canada; 8 Julius Kühn Institute (JKI) - Federal Research Centre for Cultivated Plants, Institute for Epidemiology and Pathogen Diagnostics, Braunschweig, Germany; 9 Julius Kühn Institute (JKI) - Federal Research Centre for Cultivated Plants, Institute for Crop and Soil Science, Braunschweig, Germany

**Keywords:** assembly, Eckhardt gel, genome organization, Nanopore sequencing, PFGE, protelomerase (telomere resolvase)

## Abstract

Members of the family *Rhizobiaceae* typically have multipartite genomes that are split between two or more replicons, including the chromosome and a variable number of extrachromosomal replicons (chromids and plasmids). Nearly all *Rhizobiaceae* replicons sequenced and described to date have a circular topology, except the linear chromid found in the genomes of most *Agrobacterium* spp. In this study, genomes of five non-pathogenic *Agrobacterium* strains and one plant tumourigenic *Allorhizobium* strain were fully sequenced. Surprisingly, genome analysis revealed that these six strains each carry an 80-kbp linear plasmid. Linear plasmids have so far not been identified in this bacterial family or other bacteria within the class *Alphaproteobacteria*. The ends of all six plasmids identified in this study have a hairpin structure with covalently closed ends. The plasmid sequences showed a high degree of homology, clearly indicating their common ancestry. Database searches led to the identification of additional linear plasmids in previously published *Rhizobiaceae* genome assemblies that were not previously recognized to have linear plasmids, suggesting that these replicons may be more widespread. Most likely, linear plasmids may be even more widely distributed than anticipated. Although the biological functions of the linear plasmids identified in this study remain unknown, they are associated with both non-pathogenic and plant tumourigenic *Rhizobiaceae* strains.

**Impact Statement**

The family *Rhizobiaceae* includes some remarkable and important representatives, such as plant symbiotic bacteria (rhizobia) and plant pathogenic bacteria associated with neoplasia (agrobacteria). In this study, the complete genome sequences of six *Rhizobiaceae* strains were generated and their genome organizations were examined. Strikingly, our results showed that these six strains harbour a linear plasmid. Moreover, GenBank searches suggested that linear plasmids may be even more widespread in the family *Rhizobiaceae*. Linear plasmids may go undetected in genome sequencing studies if the assemblies are not specifically examined for linear plasmids. Overall, this study provides further evidence for the extraordinary genome plasticity of members of the family *Rhizobiaceae* and expands the taxonomic range in which linear plasmids have been identified. To the best of our knowledge, this is the first report of linear plasmids in the family *Rhizobiaceae* or the class *Alphaproteobacteria*.

## Data Summary

The whole‐genome sequences have been deposited at DDBJ/ENA/GenBank under the accessions CP192696-CP192701 (Av2), CP192797‐CP192803 (rho-7.1), CP192788‐CP192796 (rho-8.1), CP192781‐CP192787 (rho-11.1), CP192774‐CP192780 (rho-13.3) and CP192764- CP192773 (rho-14.1), within the BioProjects PRJNA557463 and PRJNA1009994. The raw sequencing reads were deposited in the Sequence Read Archive under the same BioProjects PRJNA557463 and PRJNA1009994: https://www.ncbi.nlm.nih.gov/bioproject/PRJNA557463 and https://www.ncbi.nlm.nih.gov/bioproject/PRJNA1009994.

## Introduction

The *Rhizobiaceae* is a large family of Gram-negative bacteria, classified within the alphaproteobacterial order *Hyphomicrobiales* (*Rhizobiales*) [1]. This family hosts diverse bacteria occurring in various environments, including plants, soil, sediments and water [2]. The most remarkable and well-known *Rhizobiaceae* are plant symbiotic bacteria (rhizobia) that primarily belong to the genera *Rhizobium*, *Sinorhizobium*, *Allorhizobium*, *Pararhizobium*, *Neorhizobium* and *Shinella* [3], as well as plant pathogenic bacteria (agrobacteria) that are predominantly found within the genera *Agrobacterium*, *Allorhizobium* and *Rhizobium* [4,6].

 Typically, the genomes of members of the family *Rhizobiaceae* are multipartite [7], meaning that they are split between two or more large (>350 kbp) DNA fragments or replicons [8]. In other words, their genome consists of the chromosome (primary replicon) and a variable number of extrachromosomal (secondary) replicons. Taxa of the family *Rhizobiaceae* are not an exception in the bacterial world, as roughly 10% of currently fully sequenced bacterial genomes are multipartite [8]. In multipartite genomes, the chromosome is the largest replicon that carries most of the genes involved in core cellular processes, which are termed essential or core genes. Extrachromosomal replicons can be variable in their size and indispensability, leading to the emergence of classification schemes to distinguish them [8]. One such scheme classifies extrachromosomal replicons into the following four groups: second chromosome, chromid, megaplasmid and plasmid [8]. In this respect, a second chromosome is defined as a replicon that results from the splitting of the chromosome into two replicons. A chromid is a replicon that carries essential (or at least very important for survival) genes and has evolved from plasmids and, therefore, has both plasmid and chromosome characteristics [9]. Megaplasmids and plasmids carry no core genes and are, therefore, non-essential replicons, although they are vitally important for the survival of bacteria in some environments. They are distinguished based on their size, although the boundaries between megaplasmids and plasmids are arbitrary. For instance, diCenzo and Finan [8] proposed a cutoff of 350 kb, where the replicons larger than 350 kb are classified as megaplasmids, while others have pointed out that a strict size threshold across taxa is likely not appropriate [10].

Moreover, bacterial replicons can also be distinguished based on their topology (circularity or linearity). In the family *Rhizobiaceae,* nearly all replicons sequenced and described to date have a circular topology. The exception is in the genus *Agrobacterium*, whose representatives carry a linear chromid, in addition to the primary circular chromosome and a variable number of circular plasmids [11,13]. The linear chromids of the genus *Agrobacterium* have covalently closed hairpin ends, which are generated by the activity of a protelomerase (telomere resolvase) [14]. The only representative of this genus that carries no linear chromid is a recently described species, *Agrobacterium divergens*, belonging to a remote *Agrobacterium* clade [15].

 In this study, the complete genome sequences of five *Agrobacterium* strains and one *Allorhizobium* strain were generated, and their genome organizations were assessed. Unlike other members of the family *Rhizobiaceae*, our results indicated that these six strains carry a linear plasmid. Linear plasmids are generally associated with a limited number of bacteria, including some members of the class *Actinomycetes* (e.g. *Streptomyces* and *Rhodococcus*) [16], *Borrelia* [17] and *Enterobacteriaceae* (e.g. *Salmonella* and *Klebsiella*) [18,19]. To the best of our knowledge, this is the first report of linear plasmids in the family *Rhizobiaceae*, or even more broadly, within the order *Alphaproteobacteria*.

## Methods

### Bacterial strains

Six *Rhizobiaceae* strains were analysed in this study (Table 1). *Allorhizobium* sp. Av2 was isolated from a crown gall tumour on grapevine and carries a tumour-inducing (Ti) plasmid (as determined by PCR) responsible for pathogenicity on plants [20]. The five *Agrobacterium* strains were isolated from aerial crown gall tumours on rhododendron [21]. They do not carry a Ti plasmid and are non-pathogenic. However, as previously determined by analysis of their draft genomes, they carry an opine-catabolic plasmid enabling them to thrive in crown gall tumours [21]. Strains rho-7.1, rho-11.1 and rho-14.1 were identified as *Agrobacterium rosae*, while the strains rho-8.1 and rho-13.3 belong to related, but novel, as-yet undescribed species, as determined in our previous study [21]. On the other hand, strain Av2 represents a new *Allorhizobium* species, related to *Allorhizobium vitis* and *Allorhizobium amplelinum* [6] and is assigned here to the genus level (Table 1). All bacterial cultures were preserved at −80 °C in nutrient broth with 20% glycerol.

**Table 1.** *Rhizobiaceae* strains analysed in this study. All strains were isolated from crown gall tumours on plants

Strain	Species	Isolation source	Year of isolation	Reference
Av2	*Allorhizobium* sp.	Grapevine	2006	[6,20]
rho-7.1	*Agrobacterium rosae*	Rhododendron	2017	[21]
rho-8.1	*Agrobacterium* sp.	Rhododendron	2017	[21]
rho-11.1	*Agrobacterium rosae*	Rhododendron	2017	[21]
rho-13.3	*Agrobacterium* sp.	Rhododendron	2017	[21]
rho-14.1	*Agrobacterium rosae*	Rhododendron	2017	[21]

### Genomic DNA extraction

High molecular weight (HMW) genomic DNA (gDNA) was extracted from bacteria grown in nutrient broth (Difco BD, MD, USA), on nutrient agar or on Tryptone Yeast extract (TY) medium (tryptone 5 g l^−1^, yeast extract 3 g l^−1^, CaCl_2_×2H_2_O 0.9 g l^−1^ and agar 16 g l^−1^) at 28 °C for 24–48 h using a QIAGEN Genomic DNA Buffer Set (QIAGEN, Germany, cat. no. 19060) and QIAGEN genomic tip 100 G^−1^ gravity-flow, anion exchange columns (cat. no. 10243). The integrity of the HMW gDNA was roughly assessed by electrophoresis in a 0.8% agarose gel. The purity and approximate concentration of HMW gDNA were determined using a NanoDrop instrument. Additionally, the quantity of HMW gDNA was checked using a Qubit dsDNA Broad Range (BR) Assay Kit (Thermo Fisher Scientific, Waltham, MA, USA).

### Nanopore sequencing and initial data processing

For strain rho-13.3, the DNA library was constructed using a ligation sequencing kit [SQK-LSK110, Oxford Nanopore Technologies (ONT)], with no size selection, and sequenced on a MinION Mk1B with a R9.4.1 flow cell (FLO-MIN106D). The raw FAST5 files were basecalled using Guppy v6.4.6 (ONT) with a super accurate (SUP) model (dna_r9.4.1_450bps_sup.cfg) and the ‘--trim_adapters’ option. The resulting FASTQ files were filtered using Filtlong v0.2.1 (https://github.com/rrwick/Filtlong) with the options --min_length 6000 and --target_bases 1000000000 or 2000000000.

For the remaining strains, DNA libraries were prepared using ONT Native Barcoding Kit 24 V14 (SQK-NBD114.24), with no size selection, and sequenced on a MinION Mk1B with a R10.4.1 flow cell (FLO-MIN114). The raw POD5 files were basecalled using Dorado v0.7.1 (ONT) with a SUP model (dna_r10.4.1_e8.2_400bps_sup@v5.0.0). Barcode demultiplexing was also performed using Dorado. The resulting BAM files were converted to FASTQ format using SAMtools v1.18 [22]. The FASTQ files were filtered using Filtlong (with options --min_length 6000 and --keep_percent 90, in two subsequent runs), as suggested by 23 [23].

### Genome assembly, error correction and annotation

Genome assembly was performed with a hybrid approach, using both long reads generated in this study and short reads generated in our previous studies [6,21]. Primarily, we relied on the long-read-first hybrid assembly, which involved long-read assembly, followed by long-read polishing and short-read polishing. In particular, long-read assembly was primarily done using Flye v2.9.4-b1799 [24]. In some cases, additional assemblers were used, including Raven v1.8.3 [25], Miniasm/Minipolish v0.3-r179/v0.1.2 [26,28], Canu v2.2 (with option ‘genomeSize=6.5 m’) [29], Trycycler v0.5.5 [30] and/or Autocycler v0.1.0 [31]. The resulting assemblies were manually curated and processed to assess if the entire genome and all the replicons were represented. The presence of plasmids was additionally assessed by gel electrophoresis methods (see below). Long-read polishing of the resulting raw assemblies was achieved using Medaka 1.11.3 (medaka_consensus, ONT), while the subsequent short-read polishing was conducted using Polypolish v0.6.0 [32]. Automated long-read first hybrid assembly was also performed by Hybracter v0.7.3 [33], using raw non-filtered reads as an input and option ‘-c 3000000’. Moreover, for comparison and for eventual identification of small plasmids, short-read first hybrid assembly was carried out using Unicycler v0.5.1 and Spades v4.0.0 [34]. All assemblers were run with standard parameters if not otherwise stated. Finally, to correct any remaining errors, long and short reads were mapped to assemblies with minimap2 v2.28-r1209 [35] and BWA v0.7.18-r1243-dirty [36], respectively, and the resulting read alignments were visually inspected. For the examination of assembly graphs produced by some assemblers, the software Bandage v0.8.1 [37] was used. To confirm the topology of each assembled sequence, the final assemblies were analysed with assembly_topology.py (https://github.com/rrwick/Assembly-topology), which classifies read alignments at contig ends into circular (read spans both contig ends on the same strand, indicating a circular junction), hairpin (read aligns to both strands at the same contig end, consistent with a covalently closed hairpin), terminating (read ends where the contig ends, with no additional sequence beyond the boundary), clipping (read aligns up to the contig boundary, but additional bases remain unaligned [‘soft-clipped’]) or ambiguous (a read that fits more than one of the above categories, e.g. within terminal inverted repeats).

 The final assemblies were annotated using the National Center for Biotechnology Information (NCBI) Prokaryotic Genomes Annotation Pipeline (PGAP) v2024-07-18.build7555 [38]. Additionally, for annotation of the linear plasmids, eggNOG-mapper version emapper-2.1.12 [39] and the eggNOG orthology data [40] were used. For eggNOG-mapper, sequence searches were performed using diamond version 2.0.11 [41]. Moreover, to aid functional annotation of some loci, blastp comparisons against the NCBI non-redundant (nr) protein database (https://blast.ncbi.nlm.nih.gov/Blast.cgi; last accessed in April 2025) [42] were conducted.

### Sequence and phylogenetic analysis

Synteny plots were made using the pgv-mummer workflow, which is a part of the pyGenomeViz version 1.5.0 genome visualization python package (https://github.com/moshi4/pyGenomeViz). This workflow employs MUMmer version 3.23 [43] to align sequences. The sequences were aligned at the nucleotide level.

Whole-plasmid average amino acid identity (AAI) was computed using EzAAI version 1.2.3 [44], including the dependencies Prodigal version 2.6.3 [45] and mmseqs2 version 16.747c6 [46]. The minimum identity threshold for AAI calculations was set to 0.2 (20%).

 For phylogenetic analysis, a protelomerase protein multi-sequence alignment was generated using MAFFT version 7 [47]. A maximum likelihood phylogeny was inferred using IQ-TREE 1.6.12 [48] available through the IQ-TREE web server (http://iqtree.cibiv.univie.ac.at/) [49]. Model selection was conducted using IQ-TREE ModelFinder [50] based on the Bayesian information criterion [51]. Branch supports were assessed by ultrafast bootstrap analysis [52] and the SH-aLRT test [53] using 1000 replicates. The trees were visualized using FigTree version 1.4.4 (https://github.com/rambaut/figtree) and edited using Inkscape version 1.2.1 (https://inkscape.org/).

### PCR analysis

For experimental detection of the presence of the linear plasmids identified in the strains sequenced in this study, a specific primer set, consisting of linp-topo_f (5′-GCGTACTTGATCGGCTTGTT-3′) and linp-topo_r (5′-CGCCAACCTTTCGACTTCAA-3′), targeting a gene putatively encoding DNA topoisomerase I, was designed using Primer 3.0 version 4.1.0 [54]. The size of the expected amplification product is 787 bp. The PCR amplifications were performed in 25 µl reaction mixtures containing 1×OneTaq Quick-Load Buffer (New England Biolabs), 200 µM of each dNTP, 0.2 µM of each primer, 0.5 U of OneTaq Quick-Load DNA Polymerase (New England Biolabs) and 10–20 ng template DNA. The thermal profile was as follows: initial denaturation at 94 °C for 1 min, 35 cycles of denaturation at 94 °C for 30 s, annealing at 55 °C for 1 min, elongation at 68 °C for 1 min and a final extension at 68 °C for 5 min. Amplification of the 16S rRNA gene was performed for DNA samples purified from the Eckhardt-type gels (see the next paragraph), as described before [55].

### Eckhardt-type gel electrophoresis

Separation and visualization of bacterial replicons, primarily plasmids, was conducted using the modified method of Eckhardt [56]. For this purpose, we used two protocols (I and II). Protocol I was as described in our previous publication [4]. Protocol II was similar to one reported by Hynes *et al*. [57]. Electrophoresis was performed in a gel containing 0.7 % (w/v) agarose and 0.3 % (w/v) SDS in 1×TBE buffer. SDS was added to the gel after melting the agarose as a 10% solution prepared in 1×TBE buffer. Bacteria were grown in 15-ml tubes in HP medium (4 g l^−1^ peptone, 0.5 g l^−1^ yeast extract, 0.5 g l^−1^ tryptone, 0.2 g l^−1^ CaCl_2_ and 0.2 g l^−1^ Mg_2_SO_4_) on a rotary shaker (200 r.p.m.) at 28 °C for ~24 h. A 0.1–0.2 ml aliquot of bacterial culture at an optical density at 600 nm (OD_600_) of 0.3–0.5 was transferred to the microfuge tube and kept on ice. Half a millilitre of cold 0.3% (m/v) sodium lauroylsarcosinate solution (prepared in 1×TBE buffer) was added, after which the cell suspension was gently mixed by pipetting and then centrifuged at 10,000 ***g*** for 1 min. The bacterial pellet was resuspended in 20 µl of ice-cold lysis solution containing 10% (w/v) sucrose, 1 mg ml^−1^ lysozyme and 0.4 mg ml^−1^ RNase A, freshly prepared in 1×TBE buffer. The mixture was then immediately loaded into the wells of the gel. Electrophoresis was run first at room temperature at 0.4 V cm^−1^ for 30 min and then in the fridge or cold room (~4 °C) at 3.2 V cm^−1^ for 6–10 h. The gel was stained in an ethidium bromide solution (1 µg ml^−1^), and the replicons were visualized under UV light. As markers, ‘*Agrobacterium fabrum*’ C58^T^, *Allorhizobium ampelinum* S4^T^ and/or *Rhizobium rhizogenes* K84 carrying replicons of known size were used. For some gels, a Quick-Load 1 kbp Extend DNA Ladder (New England BioLabs, Inc., Ipswich, MA, USA) was also included.

### Extraction of replicon DNA separated by Eckhardt-type gel electrophoresis

DNA was recovered from the respective replicons of interest visible on the gel after their separation by the Eckhardt-type method. Briefly, bands were cut out from the Eckhardt-type gels, and the DNA was purified using Zymoclean Large Fragment DNA Recovery Kit (Zymo Research, CA, USA, cat. no. D4045) following the manufacturer’s instructions. The only modification was that the amount of agarose dissolving buffer was increased to 4–5 volumes per volume of the excised agarose gel slice. This modification was made to reduce the final concentration of SDS in the solution. SDS was present at a concentration of 0.3% (w/v) in the Eckhard-type agarose gel, while the indicated kit tolerance to this compound was ≤0.1%. To obtain a sufficient concentration of the replicon DNA, DNA from several lanes was cut out and combined before purification. The integrity of the recovered DNA was roughly assessed by electrophoresis using a 0.8% agarose gel. The purity and approximate concentration of DNA were determined using a NanoDrop instrument.

### Illumina sequencing of replicon DNA, data processing and analysis

Library preparation and sequencing were performed by Novogene GmbH (Munich, Germany). Briefly, DNA libraries were prepared with the Novogene NGS DNA Library Prep Set (cat. no. PT004), involving random DNA shearing into shorter fragments, end repair, A-tailing and ligation with Illumina adapters. The resulting fragments with adapters were size selected, PCR amplified and purified, after which they were sequenced using Illumina 150 bp paired-end sequencing on the Illumina NovaSeqX Plus platform (Illumina, CA, USA).

Adapter trimming and quality filtering of raw reads were conducted with fastp v0.23.4 [58] using default settings. The depth of coverage statistics (mean, max and min) for each replicon was assessed as follows. First, ‘clean’ reads were mapped to the corresponding complete genome sequence obtained in this study using BWA, and the resulting BAM files were sorted with SAMtools ‘sort’ command. Next, the read depth at each nucleotide position of the genome was calculated with the SAMtools ‘depth’ option. Finally, mean, max and min values for depth of coverage were calculated for each replicon using the datamash program (https://www.gnu.org/software/datamash/).

### PFGE

####  

For preparation of agarose plugs, bacteria were grown in Yeast Extract Mannitol (YEM) medium (0.5 g l^−1^ yeast extract, 5 g l^−1^ mannitol, 0.5 g l^−1^ K_2_HPO_4_, 0.2 g l^−1^ Mg_2_SO and 0.1 g l^−1^ NaCl) for 48 h at 28 °C. A 5 ml culture was centrifuged (13,000 ***g*** for 10 min), after which the pellet was washed with 500 µl of 0.5 M NaCl, centrifuged again (13,000 ***g*** for 10 min), the supernatant removed and the pellet resuspended in 500 µl of TE buffer. A 2% (w/v) low gelling temperature agarose (Sigma-Aldrich) was dissolved in TE buffer and kept in a water bath at 45 °C for about 10 min to stabilize the temperature. The 500 µl bacterial suspension in TE buffer was mixed with 500 µl of melted low gelling temperature agarose, mixed thoroughly and applied to wells (one 10-well plug mould=10 plugs of the same bacterial strain). The mixture was left at room temperature for 10 min and then incubated at 4 °C for a further 10 min to solidify. Then, the agarose plugs were pushed out from the wells into small plastic containers with a closure. Ten millilitres of TE buffer with lysozyme (Merck) (1.5 mg lysozyme/ml TE buffer) was added to each container, and the plugs were incubated overnight at 37 °C with gentle shaking (80 r.p.m.).

The next day, the buffer was removed, and 10 ml of lysis buffer [50 mM EDTA (pH 8.0), 50 mM Tris-HCl (pH 8.0) and 1% (w/v) N-lauryl sarcosine] with proteinase K (Sigma-Aldrich) (0.5 mg proteinase/ml buffer) was added. The plugs were incubated at 37 °C with gentle shaking (80 r.p.m.) for 48 h.

Afterwards, the buffer was removed, 10 ml of TE buffer was added, and the plugs were shaken (60 r.p.m.) at 37 °C for 20–30 min. The buffer was removed, replaced with fresh TE buffer, and the incubation was repeated. To inactivate proteinase K, the TE buffer was removed, replaced with TE buffer containing PMSF (Sigma-Aldrich) (0.4 mg PMSF/ml TE buffer) and incubated on a shaker with gentle mixing at 37 °C for 1 h. Then, the buffer was removed, and the plugs were washed twice with TE buffer – 10 ml at 37 °C for 20 min each, with gentle mixing. Finally, the plugs were stored in TE buffer at 4 °C until further analysis.

Gels for electrophoresis were prepared with 1% agarose in 0.5× TBE buffer. The agarose plugs were placed in wells and immobilized by overlaying with melted low gelling temperature agarose. PFGE was carried out in 0.5× TBE buffer using a CHEF-DR III system (Bio-Rad) and the following conditions: voltage 6 V cm^−1^, reorientation angle 120°, switch time 1–25 s, 5–30 s or 10–40 s, temperature 14 °C and time 22 h or 30 h.

For S1 nuclease treatment, the agar plugs were pre-incubated in 200 µl of 1× S1 buffer at room temperature for 30 min. Then, the plugs were incubated in 200 µl of fresh 1× S1 buffer containing 10 U of S1 nuclease (Thermo Fisher Scientific) and incubated at 37 °C for 20 min. The reaction was stopped by suspending the plugs in 200 µl of 0.5M EDTA. The plugs in EDTA were incubated at room temperature for 10 min. Then, the EDTA solution was substituted with 1× TE and left at room temperature for at least 30 min prior to loading the gel.

As markers, *All. ampelinum* S4^T^ carrying replicons of known size and/or ProMega-Markers Lambda Ladders (Promega) were also included.

## Results and discussion

### Whole-genome sequencing revealed the presence of a linear plasmid

A draft genome sequence of *Agrobacterium* sp. rho-13.3 was obtained in our previous study, and its phylogenetic position was elucidated [21]. As this strain formed a distinct and novel phylogenetic lineage of the genus *Agrobacterium*, which represented an outgroup to the other members of the *Agrobacterium* clade ‘rubi’, here we used ONT long-read sequencing to generate a higher quality genome assembly.

As the strain rho-13.3 was sequenced alone on an ONT flow cell, a tremendous amount of ONT data (9.4 Gbp) was generated, resulting in an excessively high depth of genome coverage (1640×, Table S1, available in the online Supplementary Material). Therefore, in this case, the data were filtered to keep ~ 1 or 2 Gbp of best reads, while still maintaining a high depth of genome coverage (~175 and ~350×) and a read length N50 of 45 or 41 kbp, respectively (Table S1).

 The genome assembly of the strain rho-13.3 comprised seven replicons (Table 2), including one large circular chromosome and a chromid with a linear topology, which is typical for this bacterial genus. Additionally, it carried multiple circular plasmids, including an opine–catabolic (OC) plasmid as characterized previously [21]. However, the assembly of two circular plasmids proved difficult for strain rho-13.3. Whole-genome assembly was initially performed using the Flye assembler with the option ‘--nano-hq’. However, assemblies differed depending on which input reads were used. For instance, when the best 1 Gbp of reads (>6 kbp read length) were used as an input, two small circular plasmids (~61 and 67 kbp) were not recovered in the resulting assembly. These plasmids were visible in the Eckhardt-type gel (protocol I, see above, Fig. S1A). One of these two plasmids was also missing when the best 2 Gbp of reads (>6 kbp read length) were used for assembly with Flye. This is not unexpected when using super-high-depth read sets with Filtlong, since the resulting subsets are enriched in longer reads and depleted in shorter ones, leading to the loss of some smaller plasmids during assembly due to insufficient coverage or representation. Therefore, the ‘--meta’ option was additionally applied, which enabled recovery of both small circular plasmids. Interestingly, in three of the four Flye assemblies, an ~80 kbp linear contig was recovered. The only exception was the Flye assembly based on the best 1 Gbp of reads without the --meta flag, from which this linear replicon was absent. The other long-read assemblers we tested (Miniasm/Minipolish and Canu) also failed to recover this replicon, except for Raven when using the best 2 Gbp of reads as input, although the ~80 kbp contig was assigned as circular in this assembly. On the other hand, assemblies generated with software that used both long and short reads (Hybracter, Unicycler and Spades) as an input did include this ~80 kbp contig. Assembly with Hybracter and Unicycler included a circularization step, but as for the Flye assemblies, an ~80 kbp contig was assigned as linear. Therefore, the authenticity and topology of this replicon in rho-13.3 were further assessed.

**Table 2.** General features of the genome sequences obtained in this study

	Strain					
	*Allorhizobium* sp. Av2	*Agrobacterium rosae* rho-7.1	*Agrobacterium* sp. rho-8.1	*Agrobacterium rosae* rho-11.1	*Agrobacterium* sp. rho-13.3	*Agrobacterium rosae* rho-14.1
Replicons (*N*)	6	7	9	7	7	10
Size (Mbp)	6.34	5.65	5.55	6.03	5.76	5.99
GC content (mol%)	57.6	56.6	56.7	56.6	56.2	56.6
Genes* (total, *N*)	5,812	5,402	5,316	5,756	5,461	5,729
CDSs* (with protein, *N*)	5,630	5,237	5,123	5,543	5,307	5,537
5 S-23S-16S rRNA operons (*N*)	4	4	4	4	4	4
Mean depth of genome coverage (×)†	353.09	487.12	602.07	379.48	1811.44	399.33
Accession number						

*Numbers based on PGAP annotation.

†Mean depth of genome coverage based on both Nanopore and Illumina data.

CDSs, coding sequences.

**Table 3.** General features of linear plasmids investigated in this study

	pLinAv2	pLin7.1	pLin8.1	pLin11.1	pLin13.3	pLin14.1
Host strain	Av2	rho-7.1	rho-8.1	rho-11.1	rho-13.3	rho-14.1
Size (bp)	78,527	80,644	76,871	83,153	80,644	80,644
Size of the terminal inverted repeat (TIR, bp)*	1,248	1,096	1,096	1,096	1,096	1,096
GC content (%)	53.73	53.83	53.78	53.93	53.82	53.83
Genes (*N*)†	88	94	92	95	93	94
Genes with hypothetical function (*N*)	58	62	61	61	61	62

*The size of the terminal inverted repeat (TIR) refers to the total length of the complementary region between the two replicon ends. Each linear replicon sequence deposited to NCBI GenBank was trimmed at the true physical end and therefore contains only one terminus of the TIR.

†Numbers based on PGAP annotation.

First, a rho-13.3 genome assembly using only short reads was performed with Spades (with option ‘--isolate’) and assembly graphs were examined. Interestingly, the ~80 kbp contig corresponding to the putative linear replicon was connected at both ends to the same end of another 1,096 bp contig. This two-contig structure is a signature of linear plasmids carrying terminal inverted repeats (TIRs) [18]. In other words, the assembly graph suggested that the shorter 1,096 bp contig represents a TIR at both ends of the ~80 kbp contig. Consistent with this interpretation, the depth of coverage for this short contig (as indicated by Spades) was double that of the longer contig. This putative linear replicon was then manually assembled, and the long Nanopore reads (>30 kbp) were mapped to the final assembly. The read alignment examination clearly showed that numerous long reads were soft-clipped over the edges of the plasmid sequence. Further examination showed that these reads continue past the end into the other strand, evidently indicating that the plasmid ends have a hairpin structure with covalently closed ends. This allowed us to identify the point where the loop occurs and the definite end of the linear plasmid. Similarly, the hairpin ends of the linear chromid, which is also carried by rho-13.3, were identified, and the replicon sequence was manually finished. Moreover, examination of long-read alignments with assembly_topology.py revealed that numerous reads classified as ‘hairpin’ mapped across both strands at the end of the plasmid (Table S2). This indicates that Nanopore reads often extend beyond the annotated plasmid end and continue into the opposite strand, consistent with a covalently closed hairpin structure. In contrast, relatively few reads terminated at the plasmid end, as would be consistent with linear plasmids without hairpins [18]. Thus, the read alignment patterns provide strong support for the hairpin-ended linear topology of these plasmids.

To identify if similar plasmids occur in other bacterial strains with publicly available genomes, blastp searches against the NCBI nr protein sequence database were performed. Not surprisingly, non-pathogenic *Agrobacterium* strains sequenced in our previous study (rho-7.1, rho-8.1, rho-11.1 and rho-14.1) [21], which were also isolated from aerial crown gall tumours on rhododendron from the same locality in Germany as strain rho-13.3, were the best hits. Surprisingly, the next-best hit was *Allorhizobium* sp. Av2, which was sequenced in a separate study from our group [6], and was isolated from a grapevine crown gall originating from Croatia. As only draft genomes were available for these additional strains, we performed ONT sequencing to generate their complete genome sequences. For strains Av2, rho-7.1, rho-8.1, rho-11.1 and rho-14.1, whole-genome ONT sequencing generated 1.2–2.4 Gbp of data per bacterial strain, enabling a high depth of genome coverage (>200×, Table S1). To improve the read length N50 for genome assembly, reads less than 6 kbp in length and 10% of the worst reads (low accuracy) were discarded, resulting in read length N50 values >15 kbp and depths of genome coverage >120× (Table S1). As is typical for *Agrobacterium* spp., genome assemblies of strains rho-7.1, rho-8.1, rho-11.1 and rho-14.1 included a large circular chromosome, a linear chromid and multiple plasmids, with the total number of replicons ranging from 6 to 10 (Table 2). On the other hand, in addition to the circular chromosome, *Allorhizobium* sp. Av2 carried a circular chromid and multiple plasmids. Among the plasmids, the *Agrobacterium* strains carried an OC plasmid characterized before [21], while the strain Av2 carried a Ti plasmid required for pathogenicity. Following analyses similar to those reported above for rho-13.3, the resulting genome assemblies confirmed the presence of a linear plasmid in all the analysed strains (Table S2).

Interestingly, *Agrobacterium rosae* strain rho-6.1, whose complete genome sequence was reported before by our group and which originated from the same locality in Germany as the other ‘rho’ strains sequenced in the current study, appeared not to carry a linear plasmid. This strain was previously sequenced using PacBio and Illumina approaches [21]. It was assembled using a long-read first approach using the ‘RS_HGAP_Assembly.3’ protocol included in SMRT Portal version 2.3.0, followed by error correction with Illumina reads. In the current study, we additionally inspected an Illumina-only Spades assembly and assembly graphs, which were also consistent with the linear plasmid being absent from this strain. Moreover, we designed primers targeting a putatively conserved gene of the linear plasmids revealed in this study and performed PCR analysis targeting a gene putatively encoding DNA topoisomerase. However, specific amplification was not observed, confirming that the strain rho-6.1 does not carry a linear plasmid.

To examine the terminal structures of the linear replicons, we compared the first and last 10 kb of each sequence by aligning the 5′ end with the reverse complement of the 3′ end. For each sequence, we recorded (i) the longest ungapped and perfectly identical block between the termini (‘exact core’), (ii) the maximal region that could be extended while maintaining ≥98% identity both globally and within 200 bp windows (High98) and (iii) the maximal region that could be extended under a more relaxed cut-off of ≥90% global identity (Tolerant90). Linear chromids generally showed short motifs (10–30 bp at the exact core level, sometimes extending beyond ~100 bp under High98) (Table S3). A TIR of similar size (25 bp) was previously identified in the C58 linear chromid [14]. An exception was observed in the rho-11.1 chromid, which contained an unusually long TIR (~33 kb under the Tolerant90 threshold), likely representing a large-scale duplication at the replicon ends. By contrast, the linear plasmids displayed longer terminal motifs, with ‘exact-core’ TIRs exceeding 500 bp in the plasmids examined (Table S3), and these TIRs showed no detectable homology to chromid TIRs. Taken together, although both linear chromids and linear plasmids identified in this study share a linear topology, they differ fundamentally in their terminal architecture, supporting independent evolutionary origins.

### Experimental validation of the presence of linear plasmids in the family *Rhizobiaceae*

In order to validate the genome assemblies, primarily the authenticity of the linear plasmid, Eckhardt-type gel electrophoresis of complete genomic DNA was conducted using two protocols. Protocol I was tailored for the separation of circular plasmids and involves a longer electrophoresis step (4 V cm^−1^ for 20 h) [4]. It allowed clear separation of the circular plasmids (61, 67, 155 and 438 kbp) of strain rho-13.3 (Fig. S1A). However, the replicon corresponding to the linear plasmid was not visible in this Eckhardt-type gel (Fig. S1A). Therefore, another slightly different Eckhardt-type protocol (protocol II) was applied, which involves a shorter electrophoretic step (3.2 V cm^−1^ for 6–10 h). This time, two additional bands were resolved for strain rho-13.3, showing faster migration speed compared to the circular plasmids (Fig. S1B). These bands may have migrated out of the end of the gel when protocol I was used. In any case, we assumed that these two bands might correspond to the linear replicons carried by this strain, the larger one being the linear chromid, and the smaller one being the linear plasmid (Fig. S1B). Likewise, a band most likely corresponding to the linear plasmid was also resolved for *Allorhizobium* sp. Av2 (Fig. S1B). *Allorhizobium* sp. Av2 does not carry a linear chromid, unlike *Agrobacterium* spp. (i.e. rho-13.3), and thus, as expected, only one band migrating faster than the circular replicons was visualized in the gel for the strain Av2 (Fig. S1B).

To estimate the size of the bands assumed to correspond to linear plasmids of strains rho-13.3 and Av2, the bands were compared to a Quick-Load 1 kb Extend DNA Ladder (New England BioLabs), which includes digested, linear DNA fragments ranging from 0.5 to 48.5 kbp. The linear plasmid bands migrated slightly slower than the largest 48.5-kbp band of the DNA ladder (Fig. S1B). Interestingly, the linear plasmids of strains rho-13.3 and Av2, with sizes of 81 and 79 kbp, respectively, migrated faster than the circular 79-kbp plasmid of the strain *All. ampelinum* S4 (Fig. S1B). The linear 79-kbp plasmid even migrated faster than the circular 44-kbp plasmid of the reference strain *R. rhizogenes* K84 (Fig. S1C). In other words, the putative linear plasmids revealed by sequencing migrated faster than expected on Eckhardt-type gels. For instance, when comparing different topological forms of the same plasmid (e.g. pUC19 of size 2.7 kbp), a supercoiled circular form migrated the fastest, followed by a linear and open circular (relaxed structure) form [59]. However, the migration speed of different plasmid forms can change with the electrophoresis conditions, such as agarose concentration, electric-field strength and ionic conditions [60,61]. Nevertheless, the standard agarose gel electrophoresis of purified DNA was used in these examples from the literature, which most likely can affect supercoiling of replicons and their mobility in the gel, compared to Eckhardt-type gel electrophoresis relying on electrophoresis of bacterial cells that are lysed directly in the gel.

**Fig. 1.**
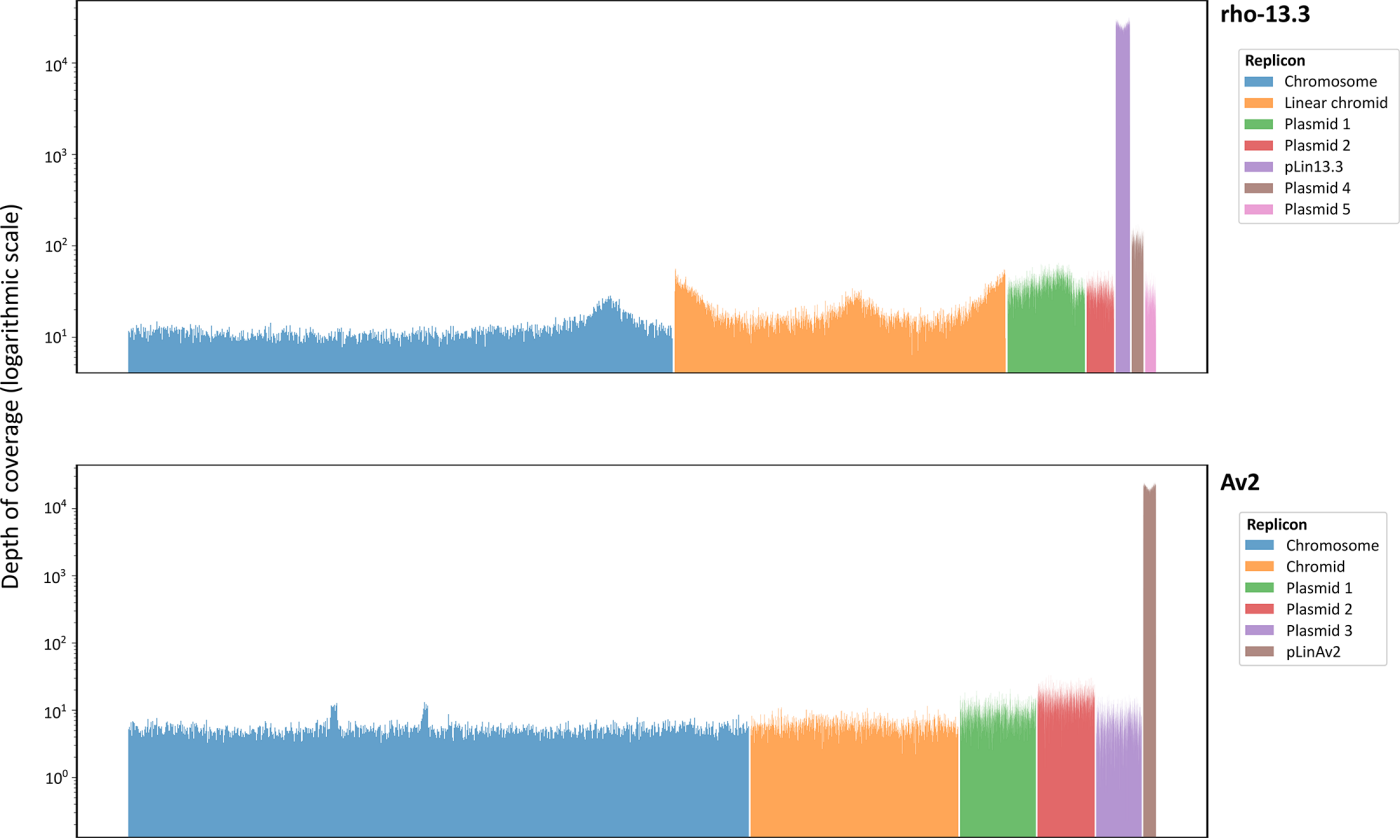
Depth of coverage along replicons. Bands corresponding to linear plasmids of strains *Agrobacterium* sp. rho-13.3 and *Allorhizobium* sp. Av2 was cut from the Eckhardt-type gel, DNA was purified and sequenced using the Illumina platform (see ‘Methods’ for more details). Plots show results for one representative DNA sample per strain (Table S2). Bars correspond to the mean coverage of 5,000 nt windows for the chromosomes, 2,000 nt windows for chromids, 500 nt windows for plasmid 1 of both strains, 200 nt windows for plasmid 2 (rho-13.3) and plasmids 2 and 3 (Av2) and 100 nt windows for pLin13.3, pLinAv2 and plasmids 4 and 5 (rho-13.3).

Taken together, Eckhardt-type gel electrophoresis suggested that the fast-migrating bands of strains rho-13.3 and Av2 might indeed correspond to the linear plasmids revealed by whole-genome sequencing. However, these data alone could not rule out that the fast-migrating bands were not either fragments of sheared DNA or circular plasmids in a compact supercoiled form. Therefore, to further verify the assignments of the fast-migrating bands as linear plasmids, they were cut from the gel and DNA was purified. Bands thought to correspond to the linear plasmids of strains *Agrobacterium* sp. rho-13.3 and *Allorhizobium* sp. Av2 was cut from two independent Eckhardt-type gels and subjected to Illumina high-throughput sequencing. The vast majority of the reads mapped back to the linear plasmid, while a small minority of reads mapped to other replicons (Fig. 1; Table S4). For example, for the band thought to correspond to the linear plasmid (pLin13.3) of strain rho-13.3, when the sequencing reads were mapped to the rho-13.3 genome, the mean depth of coverage exceeded 25,000× for pLin13.3, while it ranged from ~10 to 110× for the other replicons (Fig. 1, Table S4). The detection of a minority of DNA from other replicons is not unexpected as sheared DNA of other replicons may comigrate with the DNA of an individual replicon [62,63]. Taken together, the sequencing results strongly indicate that the fast-migrating bands indeed represent linear plasmids.

Moreover, the band presumably corresponding to the linear chromid of the strain rho-13.3 was also purified from the gel and sequenced. For this DNA sample, the mean depth of coverage reached 801× for the linear chromid, while it was <50× for other replicons, except for pLin13.3, for which it reached 233× (Fig. S2, Table S4). This band indeed seems to primarily correspond to the linear chromid DNA (most likely sheared), although it contained relatively high amounts of DNA corresponding to the linear plasmid (pLin13.3), which was resolved in close proximity in the Eckhardt-type gel (Fig. S1B).

PFGE was used to further validate the presence of linear plasmids in the six sequenced strains. For this purpose, different pulse times were used, enabling the separation of either large or small replicons. By using a pulse time of 10–40 s, it was possible to separate all plasmids of strains rho-13.3 and Av2, including the linear plasmid (Fig. S3A). Unlike Eckhardt-type gel electrophoresis, in PFGE, topology (linear vs. circular) tends not to influence the mobility of a replicon (Fig. S3). For instance, a band presumed to correspond to the pLin13.3 migrated more slowly than a band corresponding to the small circular plasmids (61 and 67 kbp) of strain rho-13.3 (Fig. S3A). As these two small circular plasmids could not be resolved by using a pulse time of 10–40 s, we confirmed the presence of these two plasmids by using a pulse time of 1–25 s, which is more suitable for the separation of smaller replicons (Fig. S3B).

 Moreover, PFGE was also performed with plugs treated with S1 nuclease. S1 nuclease targets single-stranded DNA that can occur in negatively supercoiled plasmids and converts them into the linear form [64,65]. In this respect, it has been reported that the circular and linear forms of plasmids can be distinguished because the supercoiled circular forms migrate more slowly than their corresponding linear forms [65,66]. Surprisingly, under the PFGE conditions used in this study, treated replicons migrated slightly slower than those treated with S1 nuclease (Fig. S3C). In the case of circular plasmids, S1 nuclease treatment might result in less compact, slower-migrating forms. However, it was interesting that a similar delay in mobility was also evident for the linear plasmid of the strain rho-13.3 (Fig. S3C). Linear plasmids identified in this study may be similar to the linear plasmids of *Borrelia burgdorferi*, which have covalently closed ends and contain a single-stranded loop at each end [67]. This plasmid form can be structurally more stable and compact, migrating more efficiently in the gel. An S1 nuclease might cleave the hairpin ends and likely introduce additional nicks, which could cause the linear plasmid to become less compact and to have slower migration in the PFGE gel.

### Genomic comparison of the linear plasmids

The GC content of the linear plasmids sequenced in this study ranged from 53.73 to 53.93 %, which is lower than the GC content of the chromosomes in these same strains, which range from 56.43 to 57.02% for the five *Agrobacterium* strains and 57.89% for *Allorhizobium* sp. Av2 (Tables2  3). The substantial difference in GC content between the linear plasmids and the chromosomes suggests that the linear plasmids were likely acquired more recently through horizontal gene transfer.

**Fig. 2.**
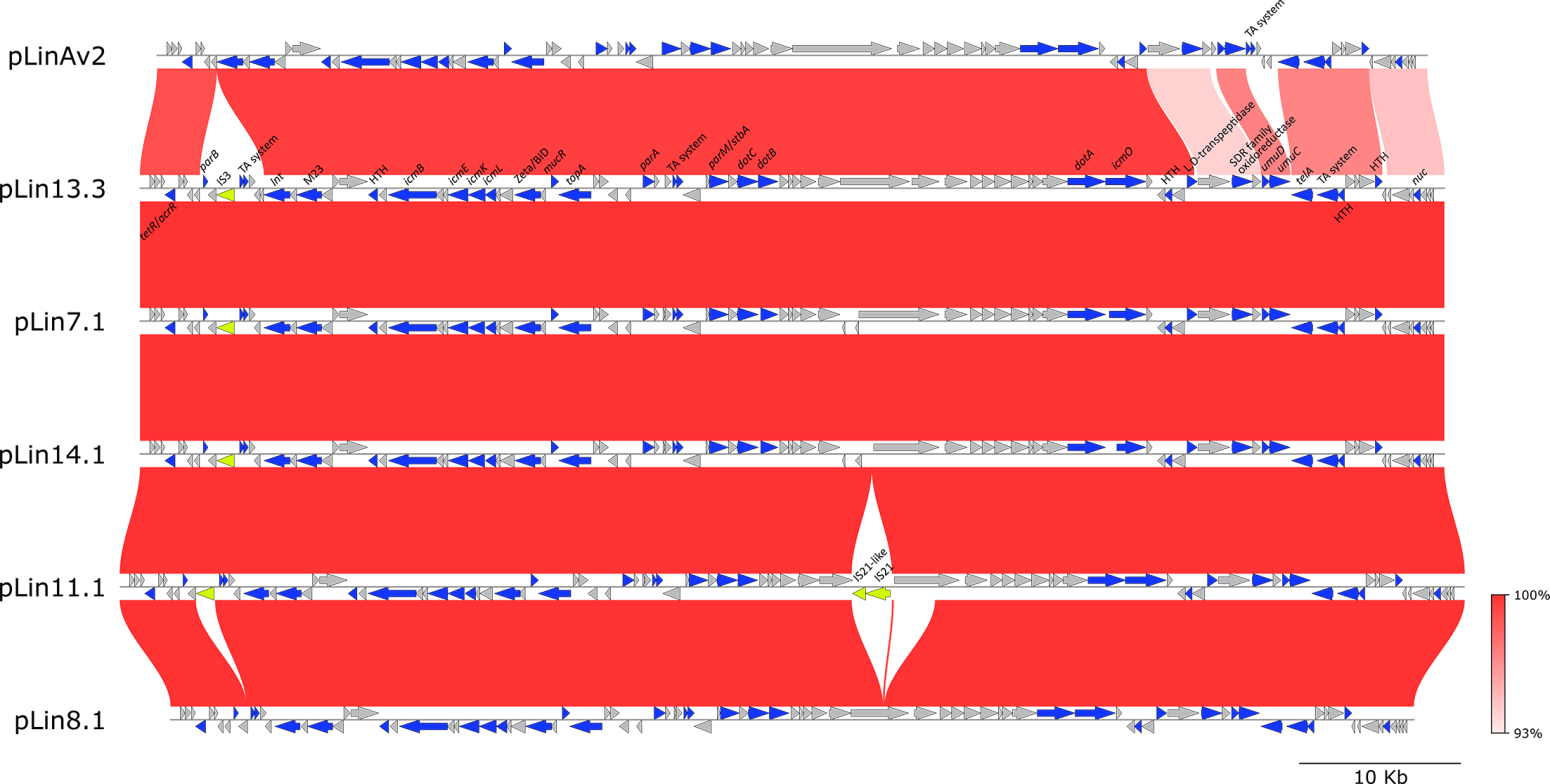
Synteny and comparative analysis of linear plasmids sequenced in this study. MUMmer alignment and visualization were performed with the ‘pgv-mummer’ workflow, which is part of the python genome visualization package ‘pyGenomeViz’ version 1.5.0 (https://github.com/moshi4/pyGenomeViz). The coloured arrows represent coding sequences (CDSs): annotated genes (blue arrows), IS elements (yellow arrows) and genes encoding hypothetical proteins (grey arrows). Gene names are indicated above the arrows only for the reference plasmid pLin13.3 (for more information, see Table S5) and for other plasmids only for the genes that are absent from pLin13.3. The red blocks connecting different gene regions of two plasmids indicate the identity between them. The darker colour indicates a higher percentage of identity.

The size of the linear plasmids ranged from 76,871 (pLin8.1) to 83,153 bp (pLin11.1) (Table 3). The differences in the size were primarily due to insertion sequence (IS) elements present in some plasmids (Fig. 2). Plasmids pLin7.1 and pLin14.1 were identical, and they differed from pLin13.3 in only one SNP. Compared to these three plasmids, plasmid pLin11.1 possessed two additional IS elements (Fig. 2). On the other hand, plasmid pLin8.1 had one fewer IS element compared to pLin7.1, pLin13.3 and pLin14.1, but contained a separate additional 2,571 bp fragment (Fig. 2). Compared to these five plasmids identified in the *Agrobacterium* strains, pLinAv2 carried by *Allorhizobium* sp. Av2 has several variations. In particular, a region including a *parB* gene, an IS3 element, a toxin–antitoxin (TA) system and several genes encoding hypothetical proteins (HPs) was absent in this plasmid, but unlike the other plasmids, it carried a different region comprising a distinct TA system as well as several genes coding for HPs (Fig. 2). Moreover, the right end of plasmid pLinAv2 showed a slightly lower degree of homology to the plasmids carried by the *Agrobacterium* strains (Fig. 2). Overall, despite the differences described above, all six linear plasmids analysed showed a high degree of homology, clearly indicating their common ancestry.

### Gene content and functional annotation of linear plasmids

The putative functions of the majority of genes carried by the linear plasmids remain unknown, and they were annotated as encoding hypothetical proteins (Table 3). In addition to the hypothetical genes, all the linear plasmids (Table 3) carried a gene putatively encoding a protelomerase (Table S5, Fig. 2), further supporting that they have a linear topology with closed hairpin ends. Unlike *Allorhizobium* sp. Av2, which does not carry additional linear replicons, the *Agrobacterium* spp. strains (Table 2) carried another gene putatively encoding a protelomerase on the linear chromids. A gene encoding a protelomerase was located on the linear chromids for some other members of the *Agrobacterium* sub-clade ‘rubi’ for which complete genome assemblies were available, i.e. *Agrobacterium vaccinii* B7.6^T^ (CP054151). This is also true for *Agrobacterium larrymoorei* CFBP 5477 (CP124734). Interestingly, in members of the *Agrobacterium* ‘biovar 1’ sub-clade, such as the strains C58^T^ and H13-3, it was reported that a protelomerase gene responsible for chromid linearity is encoded on their circular chromosome rather than on the chromid itself [13,14, 68]. Protelomerases of the linear plasmids were distinct from those associated with the linear chromids of *Agrobacterium* spp. For instance, protelomerase of pLin13.3 shared 67.3% amino acid identity with protelomerases associated with linear chromids of the same strain rho-13.3.

The linear plasmids harboured genes putatively involved in plasmid partitioning (*parA*, *parM/stbA*) and multiple TA systems putatively associated with plasmid maintenance, although they may also play roles in stress responses or phage resistance (Table S5, Fig. 2). The representative plasmid pLin13.3 encoded three type II TA families: VapBC, BrnT/BrnA and HipA (Table S5). The same three families were also found on pLin7.1, pLin8.1, pLin11.1 and pLin14.1 (Fig. 2). In contrast, although pLinAv2 carried VapBC and HipA, which are conserved across all linear plasmids, it contained a HigBA locus in which a RelE/ParE-family toxin (HigB) is located immediately upstream of a HigA antitoxin, instead of the BrnT/BrnA family found on the other linear plasmids. blastp analyses showed that HigA had its closest matches in *Rhizobiales* (up to~91% identity) with additional *Enterobacteriaceae* homologs around~75% identity (e.g. *Candidatus* Regiella), while the adjacent HigB-like toxin had close matches in *Enterobacteriaceae* (e.g. *Escherichia fergusonii*, 88% identity), consistent with previous reports of HigBA loci identified in *Enterobacteriaceae* linear plasmids [18]. Other genes potentially associated with stress tolerance or DNA damage include genes putatively encoding some components of DNA polymerase V (subunits C and D). Interestingly, DNA polymerase V is an error-prone polymerase [69], and some rhizobia encode error-prone DNA polymerases that appear to result in elevated mutation rates during legume symbiosis [70]. It is, therefore, tempting to speculate that the DNA polymerase V encoded by the linear plasmids may similarly result in elevated mutation rates during tumourigenesis. In addition, all six linear plasmids carried genes putatively encoding an apolipoprotein *N*-acyltransferase (*lnt*), a M23 family metallopeptidase (M23) and a l,d-transpeptidase (Table S5, Fig. 2) involved in lipoprotein production, lysis of bacterial cell wall peptidoglycans and peptidoglycan cross-linking, respectively. These functions might have a role in cell wall remodelling for persistence or survival under stress. A gene putatively coding for a type I DNA topoisomerase carried by these plasmids is most likely involved in relieving supercoiling of linear DNA during DNA replication and transcription (Table S5, Fig. 2).

Moreover, *dot* and *icm* genes putatively associated with the type IV secretion system (T4SS) were conserved in all six linear plasmids (Table S5, Fig. 2). In general, *dot*/*icm* genes encode a T4SS classified as type IVB (T4SS^Dot/Icm^), which is evolutionarily most closely related to conjugation systems of IncI plasmids, while a distinct type IVA resembles the VirB/VirD4 T4SS of *Agrobacterium tumefaciens* [71,72]. The T4SS^Dot/Icm^ is present in intracellular bacterial pathogens such as *Legionella pneumophila* and related bacteria, where it is crucial for virulence by translocating a large number of effectors into the eukaryotic host cell [73]. However, *dot*/*icm* gene clusters were also identified in diverse bacteria, including the genera *Burkholderia*, *Methylorubrum* and *Xanthomonas*, where they were hypothesized to play a role in conjugation [74]. Indeed, it was demonstrated that the T4SS^Dot/Icm^ of *L. pneumophila* has the capacity to transfer plasmid DNA from one cell to another [75]. Additional blast searches also revealed some homology (<53% of identity for~50% query cover) between a gene located downstream of the *icmL* gene (Zeta/BID, Fig. 2) and proteins annotated as ‘zeta toxin family proteins’ as well as ‘BID domain-containing T4SS effectors’ (Table S5). Interestingly, the BID domain is present in alphaproteobacterial toxins, relaxases and *Bartonella* effector proteins [76]. Taken together, it is tempting to speculate that the T4SS^Dot/Icm^ identified in the linear plasmids is involved in their conjugation, although we could not identify a corresponding relaxase gene or an oriT. Alternatively, considering the other described functions of T4SSs [77], it might play a role in bacterial competition by delivering toxins to competing bacteria or by injecting effector proteins into plant cells. Nevertheless, apart from the Zeta/BID gene, whose function is highly speculative, we could not identify type IV effector proteins in the linear plasmids.

### The distribution and evolution of linear plasmids

To examine whether additional organisms carried linear plasmids related to the six linear plasmids identified as part of this study, we searched GenBank using the protein sequences of the telomere resolvase and DNA topoisomerase I as queries against the nr protein database. This led to the identification of an additional six replicons/contigs that carried both a telomere resolvase and a DNA topoisomerase I, and for which raw sequencing data were available, allowing further examination (Table S6). The corresponding genes of these six newly identified strains shared low sequence identity with the telomere resolvase (~28–30%) and DNA topoisomerase I (~42–44 %) proteins of the six linear plasmids previously identified in this study. All six strains belonged to the family *Rhizobiaceae*, including one *Allorhizobium* strain, three *Rhizobium* strains and two *Agrobacterium* strains (Table S6). For the four strains for which short-read data were available (Table S6), assembly using Spades and/or Shovil was performed, and assembly graphs were examined. In all cases, the replicons carrying a telomere resolvase and a DNA topoisomerase I were connected at both ends to the same end of another short contig, a signature of plasmids with a linear topology. For the other two strains, for which only long-read data were available, we inspected the corresponding contigs and identified hairpin ends (Table S6). Overall, these results are consistent with these six new *Rhizobiaceae* replicons also representing linear plasmids.

Whole-plasmid AAI comparisons showed that the six putatively linear plasmids discovered by GenBank mining are diverse (Table S7 and Fig. S4). Nevertheless, putative linear plasmids of strains AB3 and AC44/96 were relatively closely related (AAI 91.6%), as were the putatively linear plasmids of strains NCPPB 2655, CECT 9324 and T5/73 (AAI ≥82.7 %). The six newly identified plasmids also carried *dot*/*icm* genes encoding a T4SS. Interestingly, the putative linear plasmid of strain AB3 carried a gene annotated as a relaxase/mobilization nuclease domain-containing protein (GenBank acc. no. NZ_MAVS02000024.1, locus_tag BBI12_024275). In the future, it would be interesting to obtain finished sequences of these six new putatively linear plasmids.

 As suggested by the single gene comparisons mentioned above, whole-plasmid AAI analyses confirmed that the six new, putatively linear plasmids were distantly related to the linear plasmids identified earlier in our study (Table S7 and Fig. S4). This indicates that these linear plasmids have independent evolutionary histories. Similarly, these two groups of linear plasmids formed well-separated clusters in a phylogenetic tree based on protelomerase protein sequences (Fig. S5). Interestingly, protelomerases associated with the linear plasmids sequenced in this study formed a monophyletic group intertwined within the protelomerases of the linear chromids (Fig. S5), potentially suggesting that they evolved from a protelomerase acquired from the agrobacterial linear chromids.

## Conclusion

Overall, our results are consistent with linear plasmids being acquired multiple independent times by diverse members of the family *Rhizobiaceae*. To the best of our knowledge, linear plasmids have not previously been identified in the family *Rhizobiaceae* or the class *Alphaproteobacteria* to which the family *Rhizobiaceae* belongs. The identification of linear plasmids in members of the family *Rhizobiaceae* provides further evidence of their extraordinary genome plasticity and expands the taxonomic range in which linear plasmids have been identified. Linear plasmids were identified in three *Rhizobiaceae* genera, and they were found even in published genome assemblies that were not previously recognized to have linear plasmids. These results suggest that linear plasmids may be even more widespread in the family *Rhizobiaceae* than what was detected in this study and that they may go undetected in genome sequencing studies if the assemblies are not specifically examined for linear plasmid. The linear plasmids identified in this study are carried by both non-pathogenic and tumourigenic organisms, although their biological functions remain unknown. In the future, it will be interesting to further explore the roles of these plasmids in the biology of these organisms.

## Supplementary material

10.1099/mgen.0.001537 Uncited Supplementary Material 1.

10.1099/mgen.0.001537 Uncited Supplementary Material 2.

## Abbreviations

AAI: average amino acid identity
gDNA: genomic DNA
HMW: high molecular weight
HPs: hypothetical proteins
IS: insertion sequence
nr: non-redundant
OC: opine–catabolic
ONT: Oxford Nanopore Technologies
PGAP: Prokaryotic Genomes Annotation Pipeline
SUP: super accurate
TA: toxin–antitoxin
Ti: tumour-inducing
TIRs: terminal inverted repeats
T4SS: type IV secretion system
